# A Journey into the Blue: Current Knowledge and Emerging Insights into Marine-Derived Peptaibols

**DOI:** 10.3390/md23120458

**Published:** 2025-11-28

**Authors:** Claudia Finamore, Carmen Festa, Mattia Cammarota, Simona De Marino, Maria Valeria D’Auria

**Affiliations:** Department of Pharmacy, University of Naples “Federico II”, 80131 Naples, Italy; claudia.finamore@unina.it (C.F.); carmen.festa@unina.it (C.F.); mattia.cammarota@unina.it (M.C.); madauria@unina.it (M.V.D.)

**Keywords:** marine fungi, peptides, peptaibols, biosynthesis, *Trichoderma*, bioactivity

## Abstract

Peptaibols represent a large family of membrane-active, linear fungal peptides, with variable lengths from 5 to 21 α–amino acid residues. As products of nonribosomal peptide synthetase (NRPS) biosynthetic machinery, they encompass several non-proteinogenic amino acids, particularly the Cα–tetrasubstituted residues, such as α–aminoisobutyric acid (Aib) and its homologue isovaline (Iva). Further distinctive features include an N-acyl terminus, such as an acetyl group, and a C-terminus containing an amino alcohol residue (such as phenylalaninol, leucinol, and valinol, among others), which neutralize charges at both termini and confer them a hydrophobic nature. Peptaibols not only represent the most abundant class among nonribosomal peptides, but they have also attracted continuous scientific interest due to their diverse pharmacological properties, including antimicrobial, cytotoxic, antifungal, and antiviral activities. In this review, we present for the first time the recently explored chemodiversity of fungal peptaibiotics derived from marine sources, with a particular focus on peptaibols. We discuss their distinctive structural features, chemical characterization, biosynthetic pathways, and biological activity profiles, with the aim of supporting ongoing research toward their development as potential pharmaceutical agents.

## 1. Introduction

Natural peptides have attracted remarkable attention over the past decades due to their high spectrum of biological activities, especially in the anticancer and antimicrobial fields and for their characteristics of high affinity, modular structure and low toxicity. Marine organisms and microorganisms represent one of the largest sources of peptides with high chemodiversity and a variety of biological activities [1,2].

Although many bioactive peptides, including those on the market and in clinical trials, were isolated from sponges, ascidians, and mollusks, host microorganisms often represent one powerful sustainable source of new peptide structures. Among the peptides producing microbes, surely eukaryotic marine fungi represent one of the most prolific sources of new linear and cyclic peptides with outstanding biological activities [3].

The largest family of naturally occurring linear nonribosomal peptides of fungal origin consists of a group of related metabolites known as peptaibols (an acronym for peptide–Aib–alcohol). Peptaibols are linear peptides composed of 5–21 amino acid residues. They are distinguished by a high proportion of α,α-dialkylated amino acid residues, such as 2-aminoisobutyric acid (Aib) and isovaline (Iva) [4]. Structurally, peptaibols are amphipathic, with hydrophobic side chains promoting membrane insertion and occasional polar residues, such as glutamine or serine, orienting toward membrane interfaces. At the *N*-terminus they are usually acetylated, a modification that enhances their hydrophobic character and metabolic stability. The C-terminus is typically capped with a 1,2-amino alcohol, such as leucinol or phenylalaninol, rather than a free carboxylic acid, a unique feature that inspired the term “peptaibol” (peptide + amino alcohol). Their structure–activity relationships are strongly influenced by the Aib content, which correlates with helicity, membrane activity, and antimicrobial potency, while terminal modifications affect membrane affinity, selectivity, and cytotoxic potential. Examples of well-known peptaibols, include alamethicin [5], trichorzins [6] and antiamebin [7] all of which illustrate these structural principles and their functional relevance.

Based on their chemical structures, they have been classified into two major categories: (i) peptaibols, which contain an acetylated *N*-terminus and an amide-bound amino alcohol at the *C*-terminus, representing the largest subgroup of peptaibiotics; (ii) lipopeptaibols, which are shorter and feature an acylated N-termini (C8, C10 fatty acid) and a high Gly content [8]. The term peptaibiotic is used when Aib is present, but the C-terminal is not an amino alcohol or displays an alternative structural motif. Peptaibols exhibit a variety of bioactivities, including anticancer, anti-inflammatory, or antimicrobial effects [9,10]. Extensive prior research has revealed that their mechanism of action is primarily associated with forming pores in the cellular membrane [9,11].

Several review papers covering different aspects of peptaibol chemistry and biochemistry have been published [9,12,13,14,15]. To the best of our knowledge, the growing family of marine peptaibols has never been specifically overviewed, and this review is aimed at providing information on structural diversity and biological/pharmacological activities of this subclass of marine fungal peptides.

## 2. Chemodiversity, Classification and Occurrence

**Figure 1:**
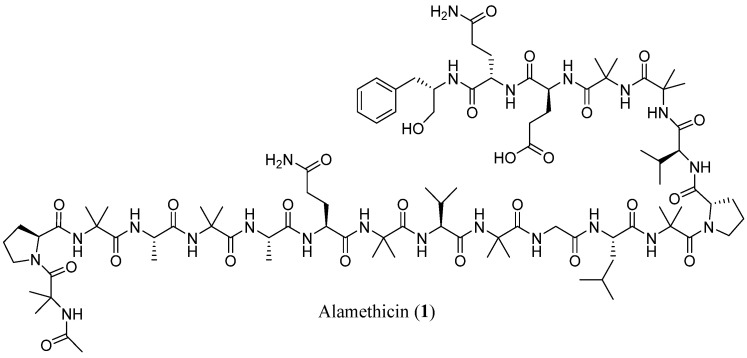
Structure of alamethicin (**1**).

Since the discovery of alamethicin (**1**, Figure 1) in the late 1960s [16] an increasing number of peptaibols has been described and summarized in offline version of Comprehensive Peptaibiotics Database [17,18], in Norine https://bioinfo.cristal.univ-lille.fr/norine/index.jsp (accessed on 25 September 2025), Protein Database https://www.rcsb.org/ (accessed on 25 September 2025), and in Antimicrobial Peptide Databases UNMC https://aps.unmc.edu (accessed on 25 September 2025) and DBAASP https://dbaasp.org/home (accessed on 25 September 2025).

**Figure 2:**
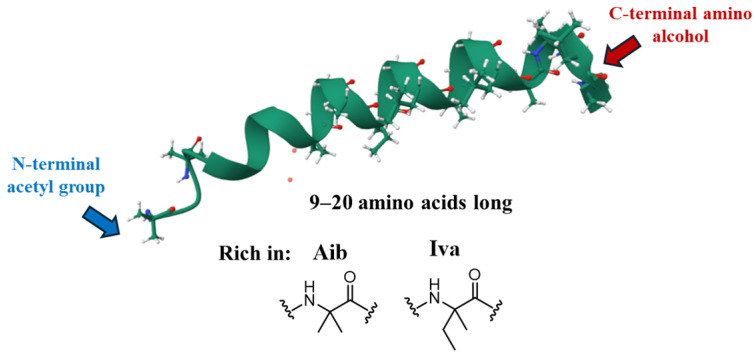
General features of peptaibols.

Peptaibols are produced as complex microheterogeneous mixtures, often comprising structurally closely related isoforms. The original classification of peptaibols was based on their length, distinguishing between “long” (17–20 residues) and “short” (11–16 residues) sequences (Figure 2). In 2001, an alternative classification into nine subfamilies (SF1–SF9) was proposed, primarily based on sequence identity and, to a lesser extent, peptide length [11]. However, this classification, based on the structures of approximately 200 peptaibols known at the time, now appears inadequate to properly categorize the more than 2000 peptaibols identified to date.

Currently, around 30 genera of filamentous fungi, mostly belonging to the order *Hypocreales*, have been disclosed as peptaibol-producing strains. Among the best-studied fungi, the members of *Trichoderma* genus, in particular the *T. viride* clade, *T. brevicompactum* clade, *T. virens*, *T. parceramosum*/*T. ghanense*, and *T. longibrachiatum* clades are the most intensively studied species for peptaibols and peptaibiotics synthesis [19,20,21,22,23,24,25].

Fungi of the *Emericellopsis* genus also produced antimicrobial peptides belonging to the peptaibol group. Compounds such as zervamicins, bergofungins, emerimicins, antiamoebin, and emericellipsins have been reported in eight different species of this genus [26,27,28]. In addition to the genera *Trichoderma* and *Emericellopsis*, species of other fungal genus, including *Acremonium*, *Paecilomyces*, *Tolypocladium*, *Clonostachys*, *Stilbella*, *Bionectria*, *Monocillium*, *Nectriopsis*, *Niesslia*, and *Sepedonium* have been identified as producers of peptaibiotics. Concerning the marine habitat, and as we’ll detail in the following sections, again *Trichoderma* peptaibols represent the large majority of the newly structures reported [8]. Other producing marine strains are *Acremonium*, *Emericellopsis*, *Stephanonectria*, and *Tolypocladium*. Worthy of note is the first report of the isolation of two related peptaibols from a bacterium: the deep sea actinomycete *Microbacterium sediminis* [29]. The presence in the genome of this new species of nonribosomal peptide synthetase modules seems to validate the bacterium as actual producer of the metabolites.

## 3. Biosynthesis

In fungal strains, peptaibol biosynthesis is encoded by large nonribosomal peptide synthetases (NRPSs) with multiple modules, each module incorporating a specific amino acid residue, often in combination with polyketide synthase (PKS) domains [12,30].

The minimal repeating unit of a NRPS contains adenylation (A), thiolation (T), and condensation (C) domains: the adenylation domain (A) is responsible of the selective loading from the cellular medium and acylation of the aminoacyl substrates, which are then linked to the phosphopantetheine prosthetic group of a thiolation domain (T) and then delivered to the condensation domain (C), where it is coupled with the upstream nascent peptide. The NRPSs responsible to produce peptaibols usually lack additional tailoring domains (epimerases and methyl transferases) responsible for the on-line synthetic modification of the loaded amino acid.

Regarding the configuration of aminoacyl residues in peptaibols, the S-configuration is usually assigned, mainly through the application of Marfey’s method. However, in some cases, peptaibols have been found to contain D-configured amino acids, as result of the specificity of certain adenylation domain, which are capable of selectively loading D-amino acids [31].

The tex1 gene from *Trichoderma virens* was the first NRPS gene linked directly to peptaibol production [32]. The synthetase contains a PKS module that is likely responsible for acylation of the N-terminal Aib residue, whereas the reduction of the carboxyl group of the C-terminal residue to the primary alcohol has been ascribed to a NAD(P)H-dependent reductase (R).

The extraordinary chemodiversity of the peptaibol family could be ascribed to several aspects of the biosynthesis that enables the production of many isoforms starting from one gene cluster.

A contributing factor is the substrate promiscuity of the adenylation domains, which can incorporate different amino acids at a given position, primarily influenced by the amino acid composition of the cultivation medium. Further diversification arises via mechanisms known as “module skipping” and “module loss” [33].

In a recent example, the genome mining of a *Trichoderma endophyticum* strain, isolated from a marine ascidian, revealed two NRPS gene clusters: one producing 11- and 14-residue peptaibols by “module skipping”, the other one responsible for 15-residue peptaibols (named “endophytins”), that arises from a 20-residue synthetase previously found in *Trichoderma* by “module loss” [30]. In few cases the “module repetition” mechanism was also observed, namely one module acts twice. The production of different isoforms from a single peptide synthetase renders difficult the possibility of producing a single peptaibol by heterologous expression in a bacterial host and so far, chemical synthesis remains the best opportunity to produce selected peptaibols in adequate yields [34]. Many synthetic efforts have been made to optimize coupling procedures that, due to the presence of α,α–disubstituted amino acids, often gave low yields. Several tailored modified analogs aimed to improve some properties such as water solubility and antibacterial activity were successful prepared and surely natural peptaibols, including those of marine origin may offer the inspiration to produce new antimicrobial molecules [35,36,37]. Regarding the industrial production, the literature indicates that peptaibols are not (so far) produced at commercial scale, at least not under standard industrial fermentation processes. Several obstacles have been identified. First, peptaibol biosynthesis often occurs under stress, or in older cultures (long cultivation times, e.g., ~15 days), while industrial fermentation tends to favor shorter growth cycles and less stressful, controlled conditions which do not favor induction of many secondary metabolites. Second, yields are generally low, and downstream purification of peptaibols from complex fungal cultures is laborious and expensive.

In 1987, Brewer et al. described an optimized process to produce trichokonin-VI, a peptaibol isolated from *Trichoderma koningii* [38,39]. From 1 kg of solid-state fermentation (SSF) culture, they obtained 146.2 mg of trichokonin-VI at a purity greater than 95%. This work represented one of the first demonstrations of high-level peptaibol production using SSF, coupled with an efficient and scalable purification method and low production cost compared with submerged fermentation, offering valuable insights into the industrial-scale production of bioactive fungal peptides.

Despite significant progress in the identification of biosynthetic gene clusters, regulatory mechanisms and the discovery of new peptaibols from marine fungi, industrial-scale production remains largely aspirational. The achievement of this goal would require optimizing culture conditions (stress, media, growth time), engineering fungal strains for higher expression, improving fermentation design (bioreactors, scale up), and developing efficient purification and safety-compliant downstream processing methods.

## 4. Bioactivity

Marine-derived peptaibols, mainly produced by fungi such as *Trichoderma* spp. isolated from marine sediments, sponges, or seaweeds, exhibit a wide range of bioactivities. These peptides are structurally similar to their terrestrial counterparts but may possess distinct sequence motifs, post- translational modifications, or amino acid substitutions that enhance their functional properties. Thanks to these unique structural adaptations, marine peptaibols represent a promising source of novel bioactive compounds, frequently demonstrating broad-spectrum activities particularly as alternative sources for antibiotic research or as new therapeutic agents [40,41]. These activities include antimicrobial [5,40,41,42,43,44,45], antifungal [43,46,47], antiviral, particularly against infection caused by the tobacco mosaic virus (notably against tobacco mosaic virus) [48,49], and antiparasitic against amoebae (*Dictyostelium* sp.) and protozoa (*Plasmodium falciparum*) [50].

In addition, their effectiveness against phytopathogenic fungi has also been explored [51,52,53,54,55].

Their amphipathic α-helical structure enables membrane disruption, causing pore formation and ion leakage. For example, short peptaibols from *Trichoderma longibrachiatum* show selective inhibition of marine *Vibrio* species and pathogenic fungi [30,56]. They effectively inhibit plant and human fungal pathogens (e.g., *Candida albicans*, *Fusarium* spp.), and their activity often surpasses that of terrestrial peptaibols, possibly due to enhanced stability or unique amino acid residues like hydroxyprolinol [56].

Previous experimental contamination studies have demonstrated that peptaibols can accumulate in filter-feeding mollusks such as *Mytilus edulis* when present in seawater as soluble compounds [57,58]. The presence of these compounds in the marine environment could lead to health risks for both shellfish and their consumers. Recent investigations have detected different peptaibols in sediments collected from a marine area dedicated to shellfish farming (Fier d’Ars, Atlantic coast, France) [59]. These sediment samples displayed high toxicity for mussel larvae in the absence of significant contaminations (metals, PCBs, HAPs, pesticides, antibiotics) or eutrophication [60].

Adaptations to the marine environment, characterized by high salinity, pressure, and unique microbial competition, may contribute to greater bioactivity and structural diversity. Their ecological significance and pharmaceutical potential warrant further exploration, especially through genome mining and chemical characterization of marine fungal isolates.

Some marine peptaibols show moderate to strong cytotoxicity against cancer cell lines, potentially via mitochondrial membrane disruption or apoptotic pathways. Pentadecaibins (from marine-derived *Trichoderma*) have been noted for selective cytotoxicity [61,62].

Moreover, peptaibols reduce microbial adhesion and biofilm formation, particularly relevant in marine antifouling applications. Their surface-active properties are useful for ecological roles and potential industrial use. Concluding, in marine ecosystems, peptaibols may function in microbial competition, symbiosis, or defense, contributing to niche adaptation of the producing organism [25,30,63] and their production can be modulated by salinity, pressure, and nutrient availability.

## 5. Marine-Origin Species

Peptaibols from marine fungi showed structural similarity with the terrestrial counterparts, and as pointed case by case, some peptaibols isolated from marine fungi were previously reported from terrestrial fungi. Also in the marine context, peptaibols were isolated as complex microhetereogeneous mixtures often differentiated by a single amino acyl substitution. This review summarizes literature data reporting the isolation or the putative annotation by mass spectrometry of about 200 new peptaibols together with those already described. Concerning some structural features, the family of marine peptaibols seems to be characterized by a preponderance of “short” peptaibols (9–11 residues), in contrast with the large majority of “long” peptaibols in terrestrial species. This finding is of interest since recent studies on short natural peptaibols or synthetic ultrashort variants disclosed enhanced activities against opportunistic pathogens [36]. The configuration of amino acyl residues in marine peptaibols was always assigned as “natural” S, except for isovaline (Iva) residues (D-configuration) [64]. The presence of “non-canonical” amino acyl residue is rare and highlighted in red in the corresponding structure drawings.

### 5.1. Peptaibols in Marine Trichoderma Species

Species of the genus *Trichoderma* are widely distributed in marine environments and like their terrestrial counterparts, they have demonstrated the ability to synthesize peptaibols when cultured under marine conditions.

In 2006 Mohamed-Benkada et al. described a methodology for identifying the sequences of short peptaibols produced by a marine strain of *Trichoderma longibrachiatum* Rifai, using an original approach based on electrospray ionization multiple-stage ion trap mass spectrometry (ESI-MS^n^-IT) [63]. Two major groups of peptaibols were identified, those with long sequences (20 amino acids) and others with short sequences (11 amino acids).

**Figure 3:**
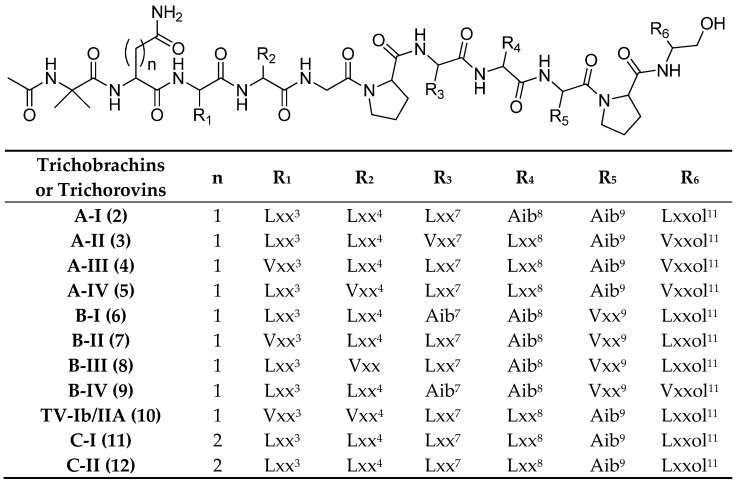
Sequences of the short peptaibols produced by marine *T. longibrachiatum* strain identified by mass spectrometry. The notations Lxx, Vxx, Lxxol and Vxxol are used when mass analysis does not allow the discrimination between Ile and Leu, Val and Iva, and their reduced forms Leu-ol and Ile-ol, Val-ol and Iva-ol, respectively. The superscript (apex) near the aa indicates the position of the residue within the sequence.

Among the nine short peptaibols identified in this study, eight were new, namely trichobrachins A I–IV (**2**–**5**, Aib^9^-Pro^10^ sequence) and trichobrachin B I–IV (**6**–**9**, Val^9^-Pro^10^ sequence), while trichorovin TV-Ib or IIA (**10**) has already been described (Figure 3) [65,66,67,68]. They were named trichobrachin A when the residue in position 2 was an Asn, and trichobrachin C when it was a Gln.

In 2007, the analysis by mass spectrometry of the peptaibol fraction from a strain of *Trichoderma longibrachiatum* isolated from the shellfish *Mytilus edulis* was reported [68]. In this work the authors focused on the sequence variability of these peptides, examining the distribution of amino acids at variable positions and exploring the relationship between hydrophobicity and cytotoxic activity against KB tumor cells. Interestingly, an exponential relationship between hydrophobicity and antiproliferative effect was observed. Thirty sequences were identified, among which twenty-one sequences were new, and nine others corresponded to peptaibols already described above and from terrestrial *Trichoderma* sp. (compounds **13**–**37**, Table 1). These peptaibols belonged to the same peptidic family based on the model Ac-Aib-xxx-xxx-xxx-Aib-Pro-xxx-xxx-Aib-Pro-xxol. Previous experimental studies have highlighted the ecological role of peptaibols in the environment [57,58].

Poirier et al. in 2007 reported the presence of long-chain peptaibols (17–20 amino acid residues) in both fresh and frozen marine sediments, as well as in *Mytilus edulis* samples, collected from Ré Island (Atlantic coast, France) [59]. Fungal strains isolated from the sediments included three belonging to the genus *Trichoderma*. It was hypothesized that these peptaibols were produced in the sediment, dissolved into the water column, and subsequently accumulated in shellfish through filtration. This study provided the first direct evidence of contamination of the marine food chain by toxic fungal metabolites. In the same year, the authors also published the first toxicological data on the embryotoxic effects of peptaibols on marine bivalve development, specifically in embryos of the Pacific oyster (*Crassostrea gigas)* [69]. The results showed that even very low concentrations of peptaibols caused significant developmental abnormalities in the embryos, such as malformations of the shell and mantle. Toxicity levels (EC_50_) for these compounds ranged between 10 and 64 nanomolar, indicating high sensitivity and highlighting the need to consider natural fungal metabolites in environmental monitoring and aquaculture.

In the context of studying the effects of exposure to natural toxins in marine environments, Ruiz et al. (2010) investigated how marine fungal metabolites might influence the neurotoxicity of domoic acid, a well-known algal neurotoxin [70]. Using a bioassay based on *Diptera larvae* (fly larvae), the researchers focused on a fungal peptaibol, specifically longibrachin A-I (**38**), a 20-amino acid compound produced by a marine-derived strain of *T. longibrachiatum* and observed as contaminant in natural marine samples [71]. This peptaibol was also reported with the name of trichokonin-VI when was isolated from *T. koningii* by Huang et al. (1996) [39] and as gliodesquin A when isolated from *Gliocladium deliquescens* by Bruckner and Przybylski (1984) [72].

**Figure 4:**
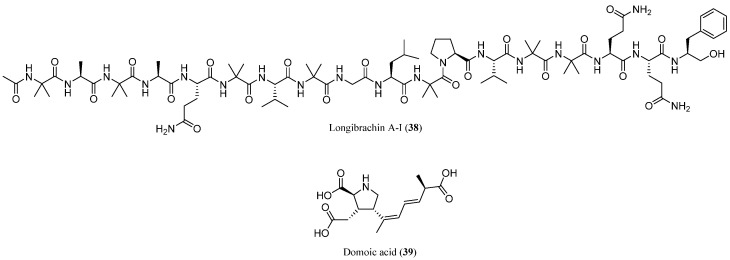
Structures of longibrachin A-I (or trichokonin VI, **38**) and domoic acid (**39**).

While longibrachin A-I (**38**) exhibited only moderate neurotoxicity on its own, the study found that when it was combined with sub-toxic doses of domoic acid (**39**, Figure 4), the resulting effect on larvae was significantly amplified. Remarkably, the presence of longibrachin A-I (**38**) enhanced domoic acid’s toxicity by up to 34.5 times. This synergistic interaction is likely due to a shared mechanism involving the disruption of ion channels and increased calcium influx in neurons, which can intensify neurotoxic effects. These findings highlight the importance of considering combined exposures to natural toxins in marine ecosystems, as such interactions may pose greater ecological and human health risks than previously recognized.

Another study on marine-derived fungal strain of *T. longibrachiatum* by Mohamed-Benkada and coworkers focused its attention on the production of long-chain peptaibols [73]. Six long-chain peptaibols were identified using advanced analytical methods such as high-performance liquid chromatography (HPLC) and tandem mass spectrometry (MS/MS); among them, three new sequences were identified: longibrachins A-0 (**40**), A-II-a (**41**), A-IV-b (**42**), Table 2. Biological assays demonstrated that these peptaibols possess significant antimicrobial activities, particularly against a range of bacterial and fungal pathogens, including strains that are relevant in clinical or agricultural context, evidencing the ecological and pharmaceutical significance of marine-derived fungi as a reservoir of bioactive natural products.

**Figure 5:**
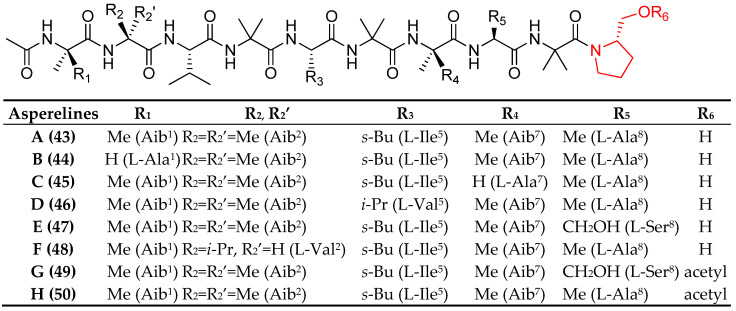
Structure of asperelines A–H (**43**–**50**). The superscript (apex) indicates the position of the residue within the sequence. The red color highlights a non-canonical feature of marine peptaibols.

Ren et al. in 2009, during their investigation on new metabolites and their biofunctions from extremophilic microorganisms, reported for the first time the isolation of a *T. asperellum* strain from the sediment of Antarctic Penguin Island [74]. Chemical investigation of its fermentation broth led to the isolation of six new peptaibols asperelines A–F (**43**–**48**, Figure 5), displaying nine amino acid residues, all of them featuring an unusual prolinol residue at the *C*-terminus and an acetylated *N*-terminus. Asperelines A–F (**43**–**48**) were tested against fungi and bacteria, but they showed only weak inhibitory activity toward the early blight pathogen *Alternaria solani*, the rice blast *Pyricularia oryzae*, and the bacteria *Staphylococcus aureus* and *Escherichia coli* with IC_50_ > 100 μg/mL and IC_90_ > 500 μg/mL, respectively.

Chen et al. (2013) reported the identification of previously known peptaibols, asperelines A (**43**) and C–F (**44**–**48**), from the marine-derived fungus *Trichoderma asperellum*, alongside two novel analogues, asperelines G and H (**49** and **50**, Figure 5), which were characterized by an acetylated C-terminus [75]. Structural elucidation was accomplished through a combination of spectroscopic techniques, single-crystal X-ray diffraction analysis, and chemical derivatization. This study provided the first structural characterization of asperelines bearing a C-terminal acetyl modification and yielded the first crystal structure determination within this class of peptaibols.

In the same year, Ren et al. revealed a remarkable degree of chemodiversity in the marine-derived fungus *Trichoderma asperellum* using ultrahigh-performance liquid chromatography coupled with electrospray ionization tandem mass spectrometry (UHPLC–ESI-MS/MS) [24]. This investigation led to the detection of notable microheterogeneity among marine-derived peptaibols and resulted in the identification of thirty-eight short-chain peptaibols, including thirty-two previously unreported analogues, likely due to their low natural abundance. These newly discovered compounds were designated as asperelines G–Z (**49**–**67**) and Z_1_–Z_13_ (**68**–**80**), in Table 3 and Table 4. While sharing a conserved structural framework, several asperelines displayed highly unusual C-terminal residues, such as proline (aspereline Z_11_, **78**) and hydroxyprolinol (aspereline Z_12_, **79**), features rarely observed in natural products. To date, peptaibols terminating in proline or hydroxyprolinol have not been reported within the broader peptaibol family, and their biological significance and functional roles remain unclear.

The study of Rangel Primo Fernandes et al. (2021) on *Trichoderma asperelloides*, an endophytic fungal strain isolated from the Amazonian aquatic plant *Victoria amazonica*, led to the tentative identification by LC-MS analysis of a family of asperelines [76]. Three new asperelines Z_14_–Z_16_ (**81**–**83**) and five known sequences, asperelines A (**43**), D (**46**), E (**47**), O (**57**) and U (**63**) were characterized by the most common *C*-terminal prolinol. Furthermore, six new asperelines (**84**–**89**), together with aspereline Z_11_ (**78**) [24], containing a C-terminal proline and likely serving as precursors to their corresponding peptaibols, were characterized (Table 4). Notably, the aspereline-containing fraction exhibited moderate activity against *Streptococcus mutans* and *Staphylococcus aureus*, and strong activity against the pathogenic bacterium *Listeria monocytogenes*, with low cytotoxicity toward Vero cells.

Carroux et al. (2013) described the analysis of an unusual series of peptaibiotics with molecular weights ranging from 1600 to 1660 Da in crude extracts from marine-derived *Trichoderma atroviride* strains [25]. This study revealed the atypical production of peptaibiotics composed of 17 amino acid residues with an unconventional C-terminus, forming a previously undescribed family of peptaibiotics (Table 5). Although the separation techniques used in this study did not fully resolve the various peptaibiotics present, mass spectrometry sequencing allowed the partial identification of 29 sequences, which were grouped into two distinct series. These two series differ at position 10, where either an Ala or a Ser residue is present. A common feature of all 17 residue peptaibols produced by this strain is the presence of an unreported C-terminal residue with the formula C_5_H_9_N_2_O_2_, indicated as C^129^. Unfortunately, the compounds were only annotated by MS^2^ analysis therefore the exact chemical nature of this residue remains undetermined. In addition to the novel 17-residue peptaibiotics, four more conventional 19-residue peptaibols were detected (Table 6).

Their sequences were compared with known 19-residue peptaibols previously described in the literature. Based on their features, sequences TA-19A-Ia/IIa/III (**119**–**121**) appear closely related to trichogin BIII, produced by *Trichoderma strigosum* [77], differing only at residue 3 (Phe replaced by Ala) and the C-terminal amino alcohol (Lxxol replaced by Pheol). The observed cytotoxicity of both the 19-residue peptaibols and the newly identified 17-residue peptaibiotics suggested that both compound types may contribute to the overall toxicity of the producing strains. Given that these peptaibiotics were consistently found in multiple marine-derived *T. atroviride* strains isolated from shellfish and their surrounding environment, the presence of this species in shellfish farming areas may pose a potential risk to shellfish health and, by extension, to consumers.

In 2013, Panizel and co-authors described the isolation and characterization of eight new peptaibols, along with four known ones, from a strain of the fungus *Trichoderma atroviride* (NF16), isolated from an axinellid sponge collected from the eastern Mediterranean coast of Israel [78].

The isolated peptaibols belong to the trichorzianine family, previously identified in *T. harzianum* and *T. atroviride*, although the peptaibol profile observed in this strain differs from those reported in earlier studies on *T. atroviride*.

**Figure 6:**
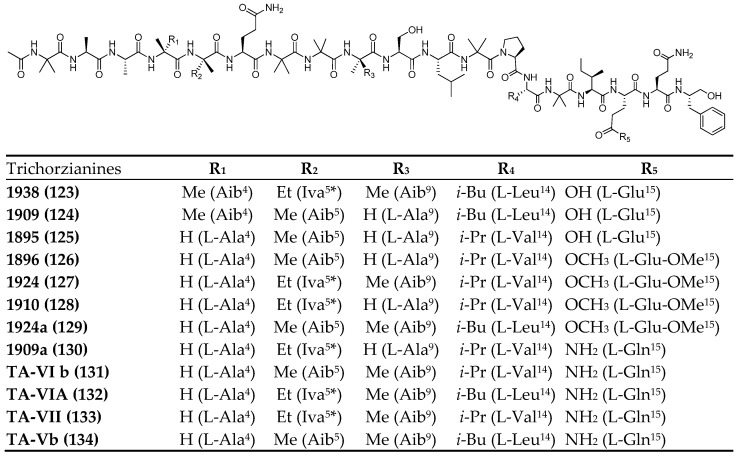
Trichorzianines (**123**–**134**) isolated from *Trichoderma atroviride* (NF16). The superscript (apex) indicates the position of the residue within the sequence. * Iva configuration was not established.

Notably, some of the newly identified compounds feature glutamic acid at position 17, whereas previously known trichorzianines (**TA**) consistently display glutamine at this position (Figure 6). Conversely, several known trichorzianines were not detected in this strain. Specifically, none of the isolated compounds contain glutamic acid at position 18 or tryptophanol (Trp-ol) at position 19. Although some fractions exhibited the presence of Trp-ol, further purification was not possible due to the limited quantity of material.

The newly identified peptaibols exhibited moderate antimicrobial activity against environmental bacteria isolated from the same Mediterranean region (MIC 12.5–200 μg/mL), except for TA-VII (**133**), which showed no activity, and TA1924 (**127**), which did not inhibit Gram-negative bacteria.

Several studies have investigated the antifungal activity of peptaibols against plant pathogens; however, only limited data are available on their activity against human pathogens [79].

**Figure 7:**
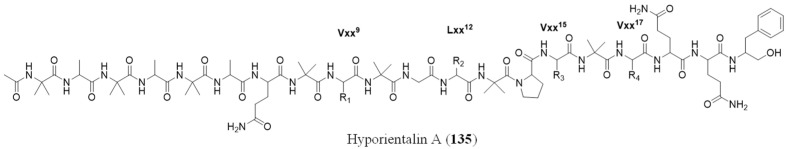
Aminoacid sequence of hyporientalin A (**135**). The notations Vxx and Lxx are used when mass analysis does not allow the discrimination between Val and Iva and Ile and Leu, respectively. The superscript indicates the position of the residue within the sequence.

In 2018, Touati et al. reported the production, purification and sequence determination of an anti-*Candida* compound, hyporientalin A (**135**, Figure 7), a peptaibol produced by a strain of *T. orientale* [80] isolated from the Mediterranean marine sponge *Cymbaxinella damicornis* [46]. Hyporientalin A (**135**) was purified and characterized by tandem mass spectrometry from this strain. Its amino acid sequence is like other known peptaibols such as trichokonin VII [39], longibrachin A-II [81], and others [74,82,83].

Hyporientalin A (**135**) exhibited broad-spectrum antibacterial and potent anti-*Candida* activity, outperforming amphotericin B against certain *Candida albicans* strains, including resistant ones [84]. Structurally, this peptaibol is rich in unusual amino acids such as Aib residues, which promote an alpha-helical conformation that enables the peptide to interact with and disrupt microbial membranes [85,86]. Its amphipathic and hydrophobic properties enhance its selective toxicity and membrane-translocating capacity. According to established guidelines for evaluating antifungal natural products, hyporientalin A (**135**) demonstrated strong in vitro antifungal activity with controlled toxicity, suggesting it is a promising candidate for development as an anti-*Candida* agent, potentially in combination with existing antifungal drugs for improved efficacy.

In 2021, van-Bohemen et al. explored the peptaibol production of the marine-derived fungal strain *Trichoderma* sp. MMS1255, as part of ongoing research into the chemodiversity of French marine *Trichoderma* species [61]. Phylogenetic analysis placed the strain within the *Trichoderma harzianum* species complex, showing a close relationship to the *T. lentiforme* lineage, though precise species identification was not possible.

**Figure 8:**
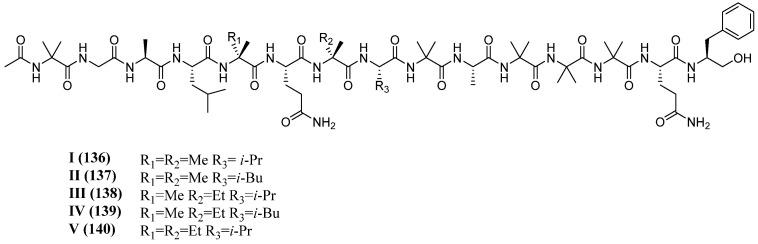
Structures of pentadecaibins **I**–**V** (**136**–**140**).

The isolated peptaibols, characterized by mass spectrometry, NMR spectroscopy, Marfey’s analysis, and circular dichroism, were named pentadecaibins I–V (**136**–**140**, Figure 8). These 15-residue peptides notably lack the typical Aib–Pro motif commonly found in many *Trichoderma*-derived peptaibols.

**Figure 9:**
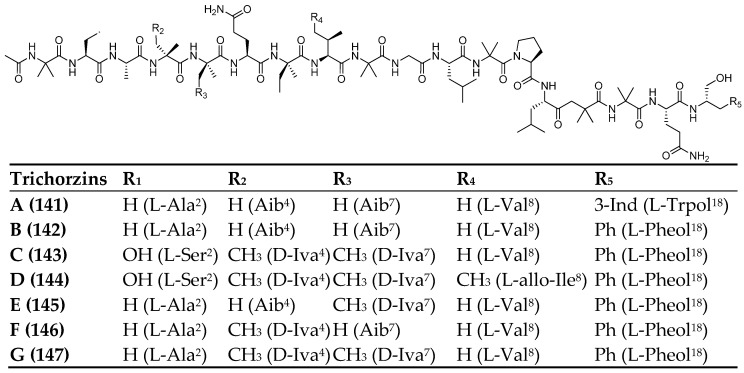
Structures of trichorzins A–G (**141**–**147**). The superscript (apex) indicates the position of the residue within the sequence.

In 2023, seven new 18-residue peptaibols, named trichorzins A–G (**141**–**147**, Figure 9) [87] were isolated from the sponge-derived fungus *Trichoderma* sp. GXIMD 01001. Their structures were fully elucidated by NMR spectroscopy, MS/MS fragmentation, Marfey’s method, and electronic circular dichroism (ECD) analysis. These peptides are characterized by a typical N-terminal acetyl group and a C-terminal α–amino alcohol, such as tryptophanol (Trp-ol) or phenylalaninol (Phe-ol), as well as a high content of Aib (α–aminoisobutyric acid) and Iva (isovaline) residues. The isolated compounds exhibited cytotoxic activity against four human cancer cell lines but showed no significant antibacterial activity. The authors proposed a preliminary structure–activity relationship, highlighting the beneficial role of the concomitant presence of Iva^4^ and Aib^7^ residues in enhancing bioactivity.

**Figure 10:**
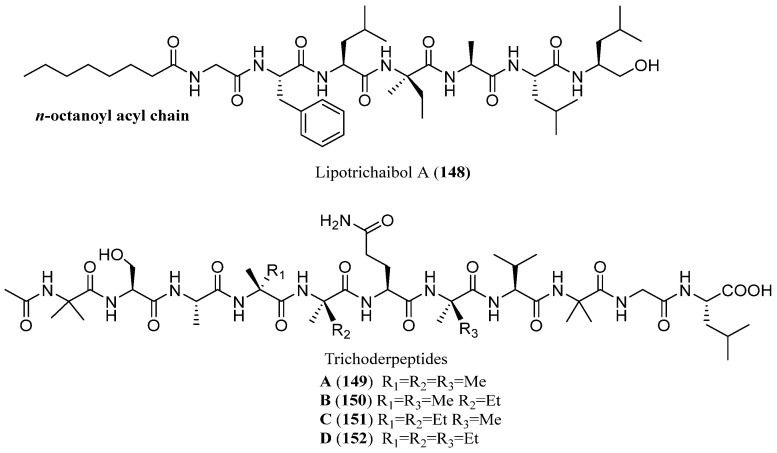
Structures of lipotrichaibol A (**148**) and trichoderpeptides A–D (**149**–**152**).

A very recent study reported the isolation of five previously undescribed peptaibiotics from the rice culture medium of the sponge-derived fungus *Trichoderma* sp. GXIMD 01001 [88]. These include one 7-mer lipopeptaibol, named lipotrichaibol A (**148**), comprising seven amino acids and an n-octyl (*n*-Oct) side chain moiety, and four linear peptaibiotics, named trichoderpeptides A–D (**149**–**152**), each consisting of eleven amino acids (Figure 10). Although numerous peptaibiotics have been identified over the past decades, the five compounds described in this study exhibit distinct structural features compared to previously reported analogues. Notably, lipotrichaibol A (**148**) contains a phenylalanine (Phe) residue at position 2, whereas most known lipopeptaibols typically have a glycine (Gly) or alanine (Ala) at this position [25,89]. Additionally, trichoderpeptides A–D (**149**–**152**) are the first 11-mer peptaibiotics reported to possess a free C-terminal carboxylic acid group. The isolated compounds were evaluated for their antiproliferative activity using CCK-8 bioassays. Among them, only lipotrichaibol A (**148**) demonstrated potent cytotoxic effects against HT-29 and DLD-1 colorectal cancer cell lines. Further in vitro assays revealed that lipotrichaibol A (**148**) significantly inhibited colony formation, induced apoptosis, and caused cell cycle arrest at the G0/G1 phase. These effects are potentially mediated through modulation of the Erk1/2 signalling pathway.

Castro et al. in 2023, reported the first identification by multilocus phylogenetic analysis (tef1-α and rpb2) of *Trichoderma endophyticum* from a marine environment in Brazil [30]. The complete genome of strain was sequenced for the first time, revealing high biosynthetic potential. Over 50 biosynthetic gene clusters (BGCs) were identified, with 66% showing no similarity to known clusters in the MIBiG (Minimum Information about a Biosynthetic Gene Cluster) database, indicating a high potential for the production of novel compounds. Notably, clusters related to tricholignans (plant growth-promoting) and clavaric acid (an anticancer triterpene) were also found.

Among the nine NRPS (nonribosomal peptide synthetases) and PKS (KS-AT-ADCP)-NRPS hybrid clusters, only one matched known cluster, suggesting that many could encode novel nonribosomal peptides. Notably, two clusters (8.3 and 19.1) displayed potential for peptaibol production:

- Cluster 8.3 showed similarity with the biosynthetic gene clusters (BGCs) related to the production of harzianins HC and hypomuricins.
- Cluster 19.1 showed similarity with (BGCs) known to produce 18–20-residues peptaibols. The actual peptaibol composition was then annotated through an integrated approach, involving molecular network, manual inspection of the MS/MS spectra and phylogenetic analysis of the adenylation domain in order to predict the incorporation of specific or variable amino acid residues in each position. The study led to the tentative identification of 21 novel 15-residue peptaibols named *Endophytins* and a smaller family of 11- and 14-residue peptaibols.

Two mechanisms of peptaibol diversification were observed:

1. Module skipping (in cluster 8.3), allowing synthesis of variable-length peptaibols;
2. Module loss (in cluster 19.1), a novel finding not previously reported in peptaibol synthesis.

Although the new compounds were not isolated, the study highlights the growing potential of integrating informatic tools such as genome-mining and molecular networking for the study of the chemodiversity of the peptaibol family.

### 5.2. Peptaibols in Marine Emericellopsis Species

Even though *Trichoderma* is the most prolific source of peptaibols, fungi of the genus *Emericellopsis* also produces antimicrobial peptides belonging to the peptaibol group.

**Figure 11:**
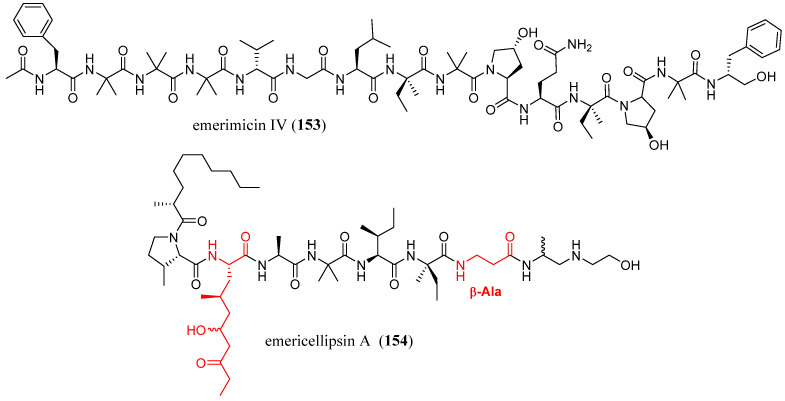
Structures of emerimicin IV (**153**) and emericellipsin A (**154**). The red color highlights a non-canonical amino acid residue of peptaibols.

Inostroza et al. (2018) conducted a screening on marine fungi collected from seabed sediments 200 m off the coast of Talcahuano Bay, Chile, with the effort to discover novel antimicrobial compounds and address the growing threat of multidrug-resistant bacteria [90]. A strain of *Emericellopsis minima* was identified, from which a unique peptaibol, emerimicin IV (**153**), was isolated (Figure 11). As expected for a peptaibol with a high content of Aib residues, emerimicin IV (**153**) exhibited significant bacteriostatic activity against clinical strains of methicillin-resistant *Staphylococcus aureus* (MRSA) and vancomycin-resistant *Enterococcus faecalis* (VRE), with minimum inhibitory concentrations (MICs) ranging from 12.5 to 100 µg/mL.

Kuvarina et al. (2022) focused their study on the production of the peptaibol emericellipsin A (**154**) by *Emericellopsis* strains (*E. alkalina*, *E.* cf. *maritima*, *E.* cf. *terricola*, *Emericellopsis* sp.), derived from soda and saline environments [91]. Emericellipsin A (EmiA, **154** Figure 11) is an antifungal peptaibol that, in previous studies, demonstrated a strong inhibitory effect against the HCT 116 and Hela cell lines [92].

Analysis of *Emericellopsis* sp. strains from the marine and terrestrial clades, isolated from chloride soils, revealed a novel form of the compound with a mass of 1032.7 Da. This variant, identified by MALDI-TOF MS/MS spectrometry, lacked a hydroxyl group and was designated as dehydroxy-emericellipsin A (dEmiA).

In this in vitro study, they demonstrated that EmiA (**154**) displayed strong inhibitory effects on the cell proliferation and viability of HCT 116 cells in dose- and time-dependent manners and induced apoptosis. These results, together with previous information regarding the effect on pathogenic fungi and cancer cells, show that lipopeptaibols EmiA (**154**), from the alkaliphilic fungus *E. alkalina* is a promising treatment alternative to licensed antifungal drugs for invasive mycosis therapy for multidrug-resistant aspergillosis and cryptococcosis.

### 5.3. Peptaibols in Marine Acremonium Species

**Figure 12:**
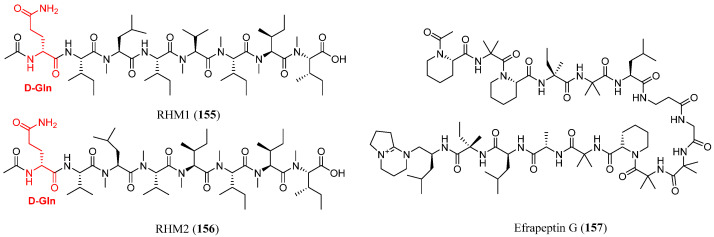
Structures of RHM1 (**155**), RHM2 (**156**) and efrapeptin G (**157**). The red color highlights a non-canonical feature observed in marine peptaibols.

To date only a few studies have examined the chemistry of marine-derived *Acremonium* strains, best known for producing the antibiotic cephalosporin C, which have yielded distinct biosynthetic products. A 2006 study [93] on a marine sponge-derived *Acremonium* strain cultivated in saltwater reported the discovery of two new octapeptides, RHM1 (**155**) and RHM2 (**156**), along with efrapeptin G (**157**), a mitochondrial ATPase inhibitor previously known only from *Tolypocladium* species (Figure 12) [94,95,96]. RHM1 (**155**) and RHM2 (**156**) are similar to previously reported N-methylated peptides, such as the dictyonamides, but are distinguished by the presence of (*R*)-glutamine, a rare feature among fungal peptides. While other fungal genera such as *Fusarium* [97], *Trichoderma* [98], and *Dendrodochium* [99] have produced peptides with (*R*)-amino acids, these were neither marine-derived nor N-methylated.

**Figure 13:**
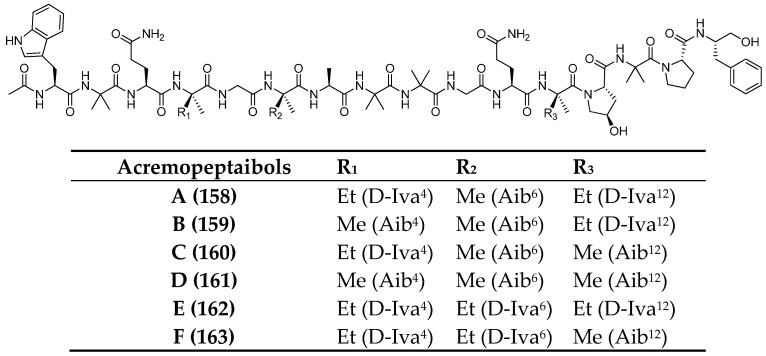
Structures of acremopeptaibols A–F (**158**–**163**). The superscript (apex) indicates the position of the residue within the sequence.

In 2021, acremopeptaibols A–F (**158**–**163**), members of a rare class of 16-residue peptaibols were isolated from cultures of the sponge-associated fungus *Acremonium* sp. IMB18-086 [100], grown on solid rice medium in the presence of autoclaved *Pseudomonas aeruginosa* (Figure 13). This cultivation condition led to up to a 20-fold increase in the production of several constitutively expressed fungal metabolites. Genomic analysis enabled the identification of a biosynthetic gene cluster (GenBank accession number MZ923510) [100] containing enough nonribosomal peptide synthetase (NRPS) modules necessary for assembling these peptaibols, thereby supporting a proposed biosynthetic pathway. Additionally, several of the isolated metabolites exhibited significant antimicrobial activity against *Staphylococcus aureus*, methicillin-resistant *S. aureus* (MRSA), *Bacillus subtilis*, and *Candida albicans*, likely through pore formation in bilayer lipid membranes. Notably, no significant cytotoxicity was observed against human cancer cell lines A549 (lung cancer) and HepG2 (hepatocellular carcinoma).

### 5.4. Peptaibols in Other Marine Species

**Figure 14:**
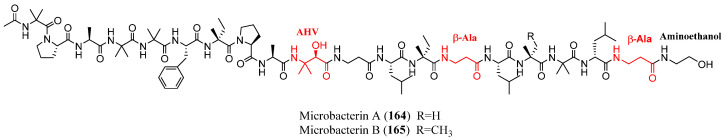
Structures of microbacterins A (**164**) and B (**165**). The red color highlights a non-canonical feature observed in marine peptaibols.

The study of the deep-sea-dwelling actinomycete *Microbacterium sediminis* sp. nov. YLB-01(T) afforded the isolation of two new peptaibols, named microbacterins A (**164**) and B (**165**) in Figure 14, by Liu et al. in 2015 [29]. The amino acid sequences were elucidated through comprehensive spectroscopic and spectrometric analysis, complemented by Marfey’s method, circular dichroism (CD), and optical rotation data for stereochemical assignments. Both compounds exhibited significant cytotoxic activity against a panel of human tumor cell lines. This represented the first report of a marine-derived actinomycete as a producer of peptaibols, containing an unusual residue of 3-amino-2-hydroxyvaline (AHV). Additionally, the C-terminal linkage via aminoethanol, observed in these compounds, is an uncommon structural feature in the peptaibol family. Although further studies are needed to clarify biosynthesis, these findings strongly suggest that deep-sea microorganisms, such as *M. sediminis*, represent a promising source of novel bioactive compounds, particularly for anticancer drug discovery.

**Figure 15:**
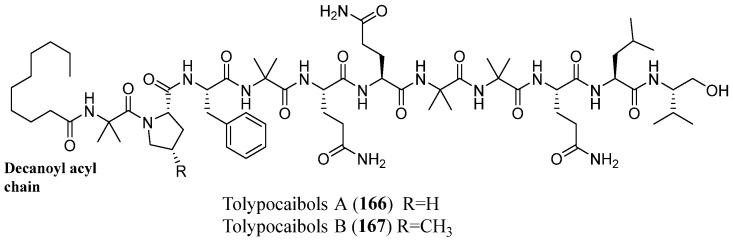
Structures of tolypocaibols A (**166**) and B (**167**).

In 2023, Morehouse et al. reported the bioassay- and LC-MS-guided fractionation of the extract of *Tolypocladium* sp. fungal endophyte from the marine alga *Spongomorpha arcta*, affording the isolation of two new lipopeptaibols, named tolypocaibols A (**166**) and B (**167**) in Figure 15, together with the known NRPS-polyketide-shikimate hybrid metabolite maximiscin [(P/M)-3)] as an inseparable mixture of interconverting atropisomers [101]. The two new compounds are 11-residue peptaibols, characterized by a C-terminal valinol and a decanoyl acyl chain at the *N-*terminus. They differ for the amino acid residue at position 2: tolypocaibol A (**166**) contains a proline (Pro-2), while tolypocaibol B (**167**) features a 4-methylproline (4-MePro), representing the first reported example of a peptaibol incorporating this nonproteinogenic amino acid. These compounds were tested for their antibacterial activities against a panel of 10 Gram-negative, seven Gram-positive, and two acid-fast bacteria. The antibacterial assays showed moderate and weak activity against Gram-positive bacteria and acid-fast bacteria *Mycobacteria tuberculosis* H37Ra (ATCC 25177) and *M. smegmatis* (ATCC 70084) while being inactive (at 128 μM) against Gram-negative bacteria.

**Figure 16:**
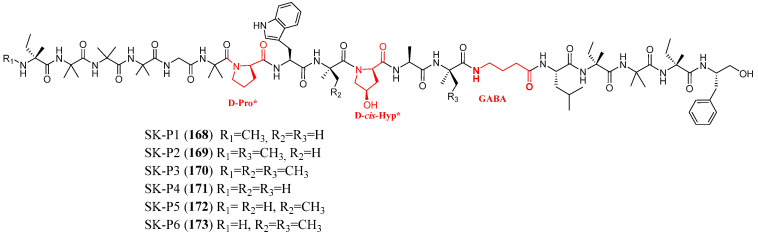
Structures of **SK-P1**–**SK-P6** (**168**–**173**). * The authors report D-configuration for the Pro and Hyp residues based on Marfey’s method, but these residues are drawn in the L-configuration in the original paper [102]. The red color highlights a non-canonical feature observed in marine peptaibols.

In 2025, Chen et al. performed the isolation of a new family of peptaibols from deep-sea fungus *Stephanonectria keithii* LZD-10-1 using biosynthetic gene clusters (BGC)-guided screening [102]. The bioinformatic analysis reveals the presence of new peptaibols. Chemical annotation of the metabolic profile using LC-HRMS/MS and GNPS molecular networking revealed several previously uncharacterized linear peptaibols, and the chromatographic separation led to the isolation of six new peptaibols, designated SK-P1 to SK-P6 (**168**–**173**, Figure 16). These compounds were identified as 18-residue peptaibols, featuring the classical high content of Aib and Iva residues, the rarely methylated or free N-terminal amine instead of the typical acylation and an γ-aminobutyric acid (GABA) residue, never previously found. All new peptaibols exhibited potent inhibitory activity against multidrug-resistant (MDR) Gram-positive bacteria, with minimum inhibitory concentration (MIC) values comparable to vancomycin and linezolid, used as positive controls. The investigation of the mechanism of action disclosed that these compounds target bacterial membrane phospholipids, specifically phosphatidylglycerol (PG) and cardiolipin (CL), leading disruption of bacterial membrane functionality and ultimately bacterial death. In addition, the authors demonstrated their efficacies against methicillin-resistant Staphylococcus aureus (MRSA) in two in vivo models, a simplified insect *Galleria mellonella* infection model and a mouse wound infection model.

To facilitate the readers, Table 7 summarizes the names and numbers of marine-derived peptides, the producing marine fungal strains, the sources from which the fungi were isolated, the biological/pharmacological activities of the bioactive peptides, and the corresponding references.

## 6. Conclusions

Compared to the study of the peptaibols from terrestrial fungal strains that began more than fifty years ago, the exploration of the chemodiversity of peptaibols from marine fungi is still in its infancy. Research in this field can, however, benefit from the advancement of modern investigation techniques such as integrated approaches involving genome-mining, mass spectrometry and molecular networking leading to a fast and more detailed overview of the complete metabolome of the species under study.

Fungal genera such as *Trichoderma*, *Emericellopsis*, and *Acremonium*, isolated from diverse marine habitats, have proven to be prolific sources of structurally diversified peptaibols, highlighting the still unexplored potential of marine ecosystems. Peptaibols from marine fungi often feature “short” 9–11 sequences, which are relatively less explored with respect to longer counterparts from terrestrial strains in terms of tridimensional behavior and ability to interact with cell membranes. The discovery of novel peptaibols from marine fungi, especially those isolated from unexplored locations and substrata, serves to unlock new opportunities for translating peptaibols into a bioactive scaffold worthy of further development in many therapeutic and agrochemical areas.

Although research has made significant strides in the characterization and understanding of the biosynthetic mechanisms of these compounds, many questions remain open, particularly regarding their precise mechanisms of action and their ecological impact, including potential health risks related to their accumulation in the marine food chain. The continued study of marine peptaibols is fundamental not only for the discovery of new pharmaceutical agents capable of addressing urgent challenges such as antibiotic resistance, but also for deepening our understanding of the complex chemical interactions that govern life in the oceans. Future efforts should focus on the isolation of new molecules, complete elucidation of their biosynthetic pathways, and rigorous evaluation of their efficacy and safety to transform the promise of these fascinating compounds into concrete therapeutic applications.

## Figures and Tables

**Table 1:** Sequences of trichobrachin peptaibols (**13**–**37**). The bold residues indicate the differences in the sequence compared with trichobrachin A-VIa (**13**), reported as reference sequence. The notations Lxx, Vxx, Lxxol and Vxxol are used when mass analysis does not allow the discrimination between Ile and Leu, Val and Iva, and their reduced forms Leu-ol and Ile-ol, Val-ol and Iva-ol, respectively. The superscript (apex) near the aa indicates the position of the residue within the sequence.

Trichobrachins	Sequence
**A-VIa (13)**	Ac-Aib^1^-Asn^2^-Vxx^3^-Vxx^4^-Aib^5^-Pro^6^-Lxx^7^-Vxx^8^-Aib^9^-Pro^10^-Vxxol^11^
**A-VIb (14)**	Ac-Aib^1^-Asn^2^-**Lxx^3^**-Vxx^4^-Aib^5^-Pro^6^-**Vxx^7^**-Vxx^8^-Aib^9^-Pro^10^-Vxxol^11^
**A-VIc (15)**	Ac-Aib^1^-Asn^2^-Vxx^3^-**Lxx**^4^-Aib^5^-Pro^6^-**Vxx^7^**-Vxx^8^-Aib^9^-Pro^10^-Vxxol^11^
**A-VId (16)**	Ac-Aib^1^-Asn^2^-Vxx^3^-Vxx^4^-Aib^5^-Pro^6^-**Vxx^7^**-**Lxx^8^**-Aib^9^-Pro^10^-Vxxol^11^
**A-VIe (17)**	Ac-Aib^1^-Asn^2^-Vxx^3^-Vxx^4^-Aib^5^-Pro^6^-**Vxx^7^**-Vxx^8^-Aib^9^-Pro^10^-Lxxol^11^
**A-VIIa (18)**	Ac-Aib^1^-Asn^2^-Val^3^-**Lxx**^4^-Aib^5^-Pro^6^-Val^7^-**Lxx^8^**-Aib^9^-Pro^10^-**Valol^11^**
**A-VIIb (19)**	Ac-Aib^1^-Asn^2^-Val^3^-**Lxx**^4^-Aib^5^-Pro^6^-**Lxx^7^**-**Val^8^**-Aib^9^-Pro^10^-**Valol^11^**
**A-VIIc (20)**	Ac-Aib^1^-Asn^2^-Val^3^-**Val^4^**-Aib^5^-Pro^6^-**Lxx^7^**-**Lxx^8^**-Aib^9^-Pro^10^-**Valol^11^**
**A-VIId (21)**	Ac-Aib^1^-Asn^2^-**Lxx^3^**-**Lxx**^4^-Aib^5^-Pro^6^-**Val^7^**-**Val^8^**-Aib^9^-Pro^10^-**Valol^11^**
**A-VIIe (22)**	Ac-Aib^1^-Asn^2^-**Lxx^3^**-**Val^4^**-Aib^5^-Pro^6^-**Val^7^**-**Lxx^8^**-Aib^9^-Pro^10^-**Valol^11^**
**A-VIIf (23)**	Ac-Aib^1^-Asn^2^-**Lxx^3^**-**Val^4^**-Aib^5^-Pro^6^-**Lxx^7^**-**Val^8^**-Aib^9^-Pro^10^-**Valol^11^**
**A-VIIg (24)**	Ac-Aib^1^-Asn^2^-**Lxx^3^**-**Val^4^**-Aib^5^-Pro^6^-**Val^7^**-**Val^8^**-Aib^9^-Pro^10^-**Leuol^11^**
**A-VIIh (25)**	Ac-Aib^1^-Asn^2^-**Val^3^**-**Lxx**^4^-Aib^5^-Pro^6^-**Val^7^**-**Val^8^**-Aib^9^-Pro^10^-**Leuol^11^**
**A-VIIi (26)**	Ac-Aib^1^-Asn^2^-**Val^3^**-**Val^4^**-Aib^5^-Pro^6^-**Lxx^7^**-**Val^8^**-Aib^9^-Pro^10^-**Leuol^11^**
**A-VIIj (27)**	Ac-Aib^1^-Asn^2^-**Val^3^**-**Val^4^**-Aib^5^-Pro^6^-**Val^7^**-**Lxx^8^**-Aib^9^-Pro^10^-**Leuol^11^**
**A-IVa (28)**	Ac-Aib^1^-Asn^2^-**Lxx^3^**-**Lxx**^4^-Aib^5^-Pro^6^-**Lxx^7^**-**Val^8^**-Aib^9^-Pro^10^-**Valol^11^**
**A-IVb (29)**	Ac-Aib^1^-Asn^2^-**Val^3^**-**Val^4^**-Aib^5^-Pro^6^-**Lxx^7^**-**Lxx^8^**-Aib^9^-Pro^10^-**Leuol^11^**
**A-IVc (30)**	Ac-Aib^1^-Asn^2^-**Val^3^**-**Lxx**^4^-Aib^5^-Pro^6^-**Val^7^**-**Lxx^8^**-Aib^9^-Pro^10^-**Leuol^11^**
**A-IVd (31)**	Ac-Aib^1^-Asn^2^-**Lxx^3^**-**Val^4^**-Aib^5^-Pro^6^-**Val^7^**-**Lxx^8^**-Aib^9^-Pro^10^-**Leuol^11^**
**A-VIIIa (32)**	Ac-Aib^1^-Asn^2^-**Lxx^3^**-**Lxx**^4^-Aib^5^-Pro^6^-**Lxx^7^**-**Lxx^8^**-Aib^9^-Pro^10^-**Valol^11^**
**A-VIIIb (33)**	Ac-Aib^1^-Asn^2^-**Lxx^3^**-**Val^4^**-Aib^5^-Pro^6^-**Lxx^7^**-**Lxx^8^**-Aib^9^-Pro^10^-**Leuol^11^**
**A-VIIIc (34)**	Ac-Aib^1^-Asn^2^-**Val^3^**-**Lxx**^4^-Aib^5^-Pro^6^-**Lxx^7^**-**Lxx^8^**-Aib^9^-Pro^10^-**Leuol^11^**
**A-VIIId (35)**	Ac-Aib^1^-Asn^2^-**Lxx^3^**-**Lxx**^4^-Aib^5^-Pro^6^-**Lxx^7^**-**Val^8^**-Aib^9^-Pro^10^-**Leuol^11^**
**A-VIIIe (36)**	Ac-Aib^1^-Asn^2^-**Lxx^3^**-**Lxx**^4^-Aib^5^-Pro^6^-**Val^7^**-**Lxx^8^**-Aib^9^-Pro^10^-**Leuol^11^**
**A-IX (37)**	Ac-Aib^1^-Asn^2^-**Lxx^3^**-**Lxx**^4^-Aib^5^-Pro^6^-**Lxx^7^**-**Lxx^8^**-Aib^9^-Pro^10^-**Leuol^11^**

**Table 2:** Sequences of the long-chain peptaibols produced by marine *T. longibrachiatum* strain identified by mass spectrometry. The notations Lxx, Vxx are used when mass analysis does not allow the discrimination between Ile and Leu, Val and Iva, and their reduced forms Leu-ol and Ile-ol, Val-ol and Iva-ol, respectively. The superscript (apex) near the aa indicates the position of the residue within the sequence.

Longibrachins	Sequence
**A-0 (40)**	Ac-Aib^1^-Ala^2^-Aib^3^-Ala^4^-Aib^5^-Ala^6^-Gln^7^-Aib^8^-Vxx^9^-Aib^10^-Gly^11^-Vxx^12^-Aib^13^-Pro^14^-Vxx^15^-Aib^16^-Aib^17^-Gln^18^-Gln^19^-Pheol^20^
**A-II-a (41)**	Ac-Aib^1^-Ala^2^-Aib^3^-Ala^4^-Aib^5^-Ala^6^-Gln^7^-Aib^8^-Vxx^9^-Aib^10^-Gly^11^-Lxx^12^-Aib^13^-Pro^14^-Vxx^15^-Aib^16^-Vxx^17^-Gln^18^-Gln^19^-Pheol^20^
**A-IV-b (42)**	Ac-Aib^1^-Ala^2^-Aib^3^-Ala^4^-Aib^5^-Aib^6^-Gln^7^-Aib^8^-Vxx^9^-Aib^10^-Gly^11^-Lxx^12^-Aib^13^-Pro^14^-Vxx^15^-Aib^16^-Vxx^17^-Gln^18^-Gln^19^-Pheol^20^

**Table 3:** Sequences of asperelines I-Z (**51**–**67**) and Z1-Z13 (**68**–**80**). The bold residues indicate the amino-acid sequence differences compared with aspereline I (**51**, reported as reference sequence). The superscript (apex) indicates the position of the residue within the sequence. The notation Lxx indicates positions where Ile/Leu are exchangeable.

Asperelines	Sequence
**I (51)**	Ac-Ala^1^-Ala^2^-Val^3^-Aib^4^-Lxx^5^-Aib^6^-Aib^7^-Ala^8^-Aib^9^-Prolinol^10^
**J (52)**	Ac-Ala^1^-**Aib^2^**-Val^3^-Aib^4^-Lxx^5^-Aib^6^-**Ala^7^**-Ala^8^-Aib^9^-Prolinol^10^
**K (53)**	Ac-**Aib^1^**-Ala^2^-Val^3^-Aib^4^-Lxx^5^-Aib^6^-**Ala^7^**-Ala^8^-Aib^9^-Prolinol^10^
**L (54)**	Ac-**Aib^1^**-**Aib^2^**-**Ala^3^**-Aib^4^-Lxx^5^-Aib^6^-Aib^7^-Ala^8^-Aib^9^-Prolinol^10^
**M (55)**	Ac-Ala^1^-**Aib^2^**-Val^3^-Aib^4^-**Val^5^**-Aib^6^-Aib^7^-Ala^8^-Aib^9^-Prolinol^10^
**N (56)**	Ac-**Aib^1^**-Ala^2^-Val^3^-Aib^4^-**Val^5^**-Aib^6^-Aib^7^-Ala^8^-Aib^9^-Prolinol^10^
**O (57)**	Ac-**Aib^1^**-**Aib^2^**-Val^3^-Aib^4^-**Val^5^**-Aib^6^-**Ala^7^**-Ala^8^-Aib^9^-Pro-ol^10^
**P (58)**	Ac-**Aib^1^**-**Aib^2^**-Val^3^-**Ala^4^**-**Val^5^**-Aib^6^-Aib^7^-Ala^8^-Aib^9^-Pro-ol^10^
**Q (59)**	Ac-**Aib^1^**-**Aib^2^**-Val^3^-Aib^4^-Lxx^5^-**Ala^6^**-Aib^7^-Ala^8^-Aib^9^-Pro-ol^10^
**R (60)**	Ac-Ala^1^-**Val^2^**-Val^3^-Aib^4^-Lxx^5^-**Ala^6^**-Aib^7^-Ala^8^-Aib^9^-Pro-ol^10^
**S (61)**	Ac-**Aib^1^**-**Aib^2^**-Val^3^-Aib^4^-Lxx^5^-Aib^6^-Aib^7^-Ala^8^-**Ala^9^**-Pro-ol^10^
**T (62)**	Ac-**Aib^1^**-**Aib^2^**-Val^3^-Aib^4^-Lxx^5^-Aib^6^-Aib^7^-Ala^8^-**Ala^9^**-Pro-ol^10^
**U (63)**	Ac-**Aib^1^**-Ala^2^-Val^3^-Aib^4^-Lxx^5^-Aib^6^-Aib^7^-Ala^8^-Aib^9^-Pro-ol^10^
**W (64)**	Ac-**Aib^1^**-Ala^2^-Val^3^-Aib^4^-Lxx^5^-Aib^6^-Aib^7^-Ala^8^-Aib^9^-Pro-ol^10^
**X (65)**	Ac-**Aib^1^**-**Aib^2^**-Val^3^-**Ala^4^**-Lxx^5^-Aib^6^-Aib^7^-Ala^8^-Aib^9^-Pro-ol^10^
**Y (66)**	Ac-**Aib^1^**-Ala^2^-Val^3^-Aib^4^-Lxx^5^-Aib^6^-**Ala^7^**-**Ser^8^**-Aib^9^-Pro-ol^10^
**Z (67)**	Ac-**Aib^1^**-**Aib^2^**-Val^3^-Aib^4^-**Val^5^**-**Ala^6^**-Aib^7^-**Ser^8^**-Aib^9^-Pro-ol^10^
**Z_1_ (68)**	Ac-**Aib^1^**-**Aib^2^**-Val^3^-Aib^4^-**Val^5^**-Aib^6^-**Ala^7^**-**Ser^8^**-Aib^9^-Pro-ol^10^
**Z_2_ (69)**	Ac-**Aib^1^**-**Val^2^**-Val^3^-Aib^4^-Lxx^5^-Aib^6^-Aib^7^-Ala^8^-**Ala^9^**-Pro-ol^10^
**Z_3_ (70)**	Ac-Ala^1^-**Val^2^**-Val^3^-Aib^4^-Lxx^5^-Aib^6^-Aib^7^-Ala^8^-Aib^9^-Pro-ol^10^
**Z_4_ (71)**	Ac-**Aib^1^**-**Aib^2^**-Val^3^-Aib^4^-Lxx^5^-Aib^6^-Aib^7^-Ala^8^-Aib^9^-Pro-ol^10^
**Z_5_ (72)**	Ac-**Aib^1^**-**Aib^2^**-Val^3^-Aib^4^-Lxx^5^-Aib^6^-**Ser^7^**-Ala^8^-Aib^9^-Pro-ol^10^
**Z_6_ (73)**	Ac-**Aib^1^**-**Aib^2^**-Val^3^-Aib^4^-Lxx^5^-**Ala^6^**-Aib^7^-**Ser^8^**-Aib^9^-Pro-ol^10^
**Z_7_ (74)**	Ac-**Aib^1^**-**Aib^2^**-Val^3^-Aib^4^-Lxx^5^-Aib^6^-**Ala^7^**-**Ser^8^**-Aib^9^-Pro-ol^10^
**Z_8_ (75)**	Ac-**Aib^1^**-Ala^2^-Val^3^-Aib^4^-Lxx^5^-Aib^6^-Aib^7^-**Ser^8^**-Aib^9^-Pro-ol^10^
**Z_9_ (76)**	Ac-**Aib^1^**-**Aib^2^**-Val^3^-Aib^4^-**Val^5^**-Aib^6^-Aib^7^-**Ser^8^**-Aib^9^-Pro-ol^10^
**Z_10_ (77)**	Ac-**Aib^1^**-**Aib^2^**-Val^3^-Aib^4^-Lxx^5^-Ser**Val^6^**-Aib^7^-Ala^8^-Aib^9^-Pro-ol^10^
**Z_11_ (78)**	Ac-**Aib^1^**-**Aib^2^**-Val^3^-Aib^4^-Lxx^5^-Aib^6^-Aib^7^-Ala^8^-Aib^9^-**Pro^10^**
**Z_12_ (79)**	Ac-**Aib^1^**-**Aib^2^**-Val^3^-Aib^4^-Lxx^5^-Aib^6^-Aib^7^-Ala^8^-Aib^9^-**Hyp-ol^10^**
**Z_13_ (80)**	Ac-**Aib^1^**-**Val^2^**-Val^3^-Aib^4^-Lxx^5^-Aib^6^-Aib^7^-**Ser^8^**-Aib^9^-Pro-ol^10^

**Table 4:** Sequences of asperelines Z_14_–Z_22_ (**81**–**89**). The notation Lxx indicates positions where Ile/Leu are exchangeable. The superscript (apex) indicates the position of the residue within the sequence.

Asperelines	Sequence
**Z_14_ (81)**	Ac-Aib^1^-Ala^2^-Val^3^-Aib^4^-Lxx^5^-Aib^6^-Aib^7^-Gly^8^-Aib^9^-Pro-ol^10^
**Z_15_ (82)**	Ac-Aib^1^-Aib^2^-Val^3^-Aib^4^-Val^5^-Aib^6^-Aib^7^-Gly^8^-Aib^9^-Pro-ol^10^
**Z_16_ (83)**	Ac-Aib^1^-Aib^2^-Val^3^-Aib^4^-Lxx^5^-Aib^6^-Aib^7^-Gly^8^-Aib^9^-Pro-ol^10^
**Z_17_ (84)**	Ac-Aib^1^-Aib^2^-Val^3^-Aib^4^-Lxx^5^-Aib^6^-Aib^7^-Gly^8^-Aib^9^-Pro^10^
**Z_18_ (85)**	Ac-Aib^1^-Ala^2^-Val^3^-Aib^4^-Lxx^5^-Aib^6^-Aib^7^-Ala^8^-Aib^9^-Pro^10^
**Z_19_ (86)**	Ac-Aib^1^-Ala^2^-Val^3^-Aib^4^-Lxx^5^-Aib^6^-Aib^7^-Ala^8^-Aib^9^-Pro^10^
**Z_20_ (87)**	Ac-Aib^1^-Aib^2^-Val^3^-Aib^4^-Val^5^-Aib^6^-Aib^7^-Ala^8^-Aib^9^-Pro^10^
**Z_21_ (88)**	Ac-Aib^1^-Ala^2^-Val^3^-Aib^4^-Lxx^5^-Aib^6^-Aib^7^-Ser^8^-Aib^9^-Pro^10^
**Z_22_ (89)**	Ac-Aib^1^-Aib^2^-Val^3^-Aib^4^-Val^5^-Aib^6^-Aib^7^-Ser^8^-Aib^9^-Pro^10^

**Table 5:** Sequences of trichorzianines (**90**–**118**). The notations Vxx and Lxx indicate positions where Val/Iva and Ile/Leu are exchangeable. The superscript (apex) indicates the position of the residue within the sequence.

Trichorzianines (TA)	Sequence
**TA-17A-I a (90)**	Ac-Aib^1^-Ala^2^-Ala^3^-Ala^4^-Aib^5^-Gln^6^-Aib^7^-Aib^8^-Aib^9^-Ala^10^-Lxx^11^-Aib^12^-Pro^13^-Vxx^14^-Aib^15^-Lxx^16^-[C^129^]^17^
**TA-17A-I b (91)**	Ac-Aib^1^-Ala^2^-Ala^3^-Aib^4^-Ala^5^-Gln^6^-Aib^7^-Aib^8^-Aib^9^-Ala^10^-Lxx^11^-Aib^12^-Pro^13^-Vxx^14^-Aib^15^-Lxx^16^-[C^129^]^17^
**TA-17A-I c (92)**	Ac-Ala^1^-Ala^2^-Ala^3^-Aib^4^-Aib^5^-Gln^6^-Aib^7^-Aib^8^-Aib^9^-Ala^10^-Lxx^11^-Aib^12^-Pro^13^-Vxx^14^-Aib^15^-Lxx^16^-[C^129^]^17^
**TA-17A-I d (93)**	Ac-Aib^1^-Ala^2^-Ala^3^-Aib^4^-Aib^5^-Gln^6^-Aib^7^-Aib^8^-Aib^9^-Ala^10^-Lxx^11^-Aib^12^-Pro^13^-Vxx^14^-Aib^15^-Vxx^16^-[C^129^]^17^
**TA-17A-I e (94)**	Ac-Aib^1^-Ala^2^-Ala^3^-Ala^4^-Vxx^5^-Gln^6^-Aib^7^-Aib^8^-Aib^9^-Ala^10^-Lxx^11^-Aib^12^-Pro^13^-Vxx^14^-Aib^15^-Vxx^16^-[C^129^]^17^
**TA-17A-II a (95)**	Ac-Aib^1^-Ala^2^-Ala^3^-Aib^4^-Aib^5^-Gln^6^-Aib^7^-Aib^8^-Aib^9^-Ala^10^-Lxx^11^-Aib^12^-Pro^13^-Vxx^14^-Aib^15^-Lxx^16^-[C^129^]^17^
**TA-17A-II b (96)**	Ac-Aib^1^-Ala^2^-Ala^3^-Ala^4^-Vxx^5^-Gln^6^-Aib^7^-Aib^8^-Aib^9^-Ala^10^-Lxx^11^-Aib^12^-Pro^13^-Vxx^14^-Aib^15^-Lxx^16^-[C^129^]^17^
**TA-17A-II c (97)**	Ac-Ala^1^-Ala^2^-Ala^3^-Aib^4^-Vxx^5^-Gln^6^-Aib^7^-Aib^8^-Aib^9^-Ala^10^-Lxx^11^-Aib^12^-Pro^13^-Vxx^14^-Aib^15^-Lxx^16^-[C^129^]^17^
**TA-17A-II d (98)**	Ac-Aib^1^-Ala^2^-Ala^3^-Ala^4^-Aib^5^-Gln^6^-Aib^7^-Aib^8^-Aib^9^-Ala^10^-Lxx^11^-Aib^12^-Pro^13^-Lxx^14^-Aib^15^-Lxx^16^-[C^129^]^17^
**TA-17A-II e (99)**	Ac-Ala^1^-Ala^2^-Ala^3^-Aib^4^-Aib^5^-Gln^6^-Aib^7^-Aib^8^-Aib^9^-Ala^10^-Lxx^11^-Aib^12^-Pro^13^-Lxx^14^-Aib^15^-Lxx^16^-[C^129^]^17^
**TA-17A-II f (100)**	Ac-Aib^1^-Ala^2^-Ala^3^-Aib^4^-Vxx^5^-Gln^6^-Aib^7^-Aib^8^-Aib^9^-Ala^10^-Lxx^11^-Aib^12^-Pro^13^-Vxx^14^-Aib^15^-Vxx^16^-[C^129^]^17^
**TA-17A-III a (101)**	Ac-Aib^1^-Ala^2^-Ala^3^-Aib^4^-Vxx^5^-Gln^6^-Aib^7^-Aib^8^-Aib^9^-Ala^10^-Lxx^11^-Aib^12^-Pro^13^-Vxx^14^-Aib^15^-Lxx^16^-[C^129^]^17^
**TA-17A-III b (102)**	Ac-Aib^1^-Ala^2^-Ala^3^-Aib^4^-Aib^5^-Gln^6^-Aib^7^-Aib^8^-Aib^9^-Ala^10^-Lxx^11^-Aib^12^-Pro^13^-Lxx^14^-Aib^15^-Lxx^16^-[C^129^]^17^
**TA-17A-III c (103)**	Ac-Aib^1^-Ala^2^-Ala^3^-Ala^4^-Vxx^5^-Gln^6^-Aib^7^-Aib^8^-Aib^9^-Ala^10^-Lxx^11^-Aib^12^-Pro^13^-Lxx^14^-Aib^15^-Lxx^16^-[C^129^]^17^
**TA-17A-III d (104)**	Ac-Ala^1^-Ala^2^-Ala^3^-Aib^4^-Vxx^5^-Gln^6^-Aib^7^-Aib^8^-Aib^9^-Ala^10^-Lxx^11^-Aib^12^-Pro^13^-Lxx^14^-Aib^15^-Lxx^16^-[C^129^]^17^
**TA-17A-IV a (105)**	Ac-Aib^1^-Ala^2^-Ala^3^-Aib^4^-Vxx^5^-Gln^6^-Aib^7^-Aib^8^-Aib^9^-Ala^10^-Lxx^11^-Aib^12^-Pro^13^-Lxx^14^-Aib^15^-Lxx^16^-[C^129^]^17^
**TA-17S-I a (106)**	Ac-Aib^1^-Ala^2^-Ala^3^-Aib^4^-Aib^5^-Gln^6^-Aib^7^-Aib^8^-Aib^9^-Ser^10^-Lxx^11^-Aib^12^-Pro^13^-Vxx^14^-Aib^15^-Lxx^16^-[C^129^]^17^
**TA-17S-I b (107)**	Ac-Ala^1^-Aib^2^-Ala^3^-Ala^4^-Vxx^5^-Gln^6^-Aib^7^-Aib^8^-Aib^9^-Ser^10^-Lxx^11^-Aib^12^-Pro^13^-Vxx^14^-Aib^15^-Lxx^16^-[C^129^]^17^
**TA-17S-I c (108)**	Ac-Aib^1^-Ala^2^-Ala^3^-Ala^4^-Vxx^5^-Gln^6^-Aib^7^-Aib^8^-Aib^9^-Ser^10^-Lxx^11^-Aib^12^-Pro^13^-Vxx^14^-Aib^15^-Lxx^16^-[C^129^]^17^
**TA-17S-I d (109)**	Ac-Ala^1^-Ala^2^-Ala^3^-Aib^4^-Vxx^5^-Gln^6^-Aib^7^-Aib^8^-Aib^9^-Ser^10^-Lxx^11^-Aib^12^-Pro^13^-Vxx^14^-Aib^15^-Lxx^16^-[C^129^]^17^
**TA-17S-I e (110)**	Ac-Aib^1^-Ala^2^-Ala^3^-Aib^4^-Aib^5^-Gln^6^-Aib^7^-Aib^8^-Aib^9^-Ser^10^-Lxx^11^-Aib^12^-Pro^13^-Lxx^14^-Aib^15^-Vxx^16^-[C^129^]^17^
**TA-17S-I f (111)**	Ac-Ala^1^-Aib^2^-Ala^3^-Ala^4^-Vxx^5^-Gln^6^-Aib^7^-Aib^8^-Aib^9^-Ser^10^-Lxx^11^-Aib^12^-Pro^13^-Lxx^14^-Aib^15^-Vxx^16^-[C^129^]^17^
**TA-17S-II a (112)**	Ac-Aib^1^-Ala^2^-Ala^3^-Aib^4^-Vxx^5^-Gln^6^-Aib^7^-Aib^8^-Aib^9^-Ser^10^-Lxx^11^-Aib^12^-Pro^13^-Vxx^14^-Aib^15^-Lxx^16^-[C^129^]^17^
**TA-17S-II b (113)**	Ac-Aib^1^-Ala^2^-Ala^3^-Aib^4^-Aib^5^-Gln^6^-Aib^7^-Aib^8^-Aib^9^-Ser^10^-Lxx^11^-Aib^12^-Pro^13^-Lxx^14^-Aib^15^-Lxx^16^-[C^129^]^17^
**TA-17S-II c (114)**	Ac-Aib^1^-Ala^2^-Ala^3^-Ala^4^-Vxx^5^-Gln^6^-Aib^7^-Aib^8^-Aib^9^-Ser^10^-Lxx^11^-Aib^12^-Pro^13^-Lxx^14^-Aib^15^-Lxx^16^-[C^129^]^17^
**TA-17S-II d (115)**	Ac-Ala^1^-Ala^2^-Ala^3^-Aib^4^-Vxx^5^-Gln^6^-Aib^7^-Aib^8^-Aib^9^-Ser^10^-Lxx^11^-Aib^12^-Pro^13^-Lxx^14^-Aib^15^-Lxx^16^-[C^129^]^17^
**TA-17S-III a (116)**	Ac-Aib^1^-Ala^2^-Ala^3^-Aib^4^-Vxx^5^-Gln^6^-Aib^7^-Aib^8^-Aib^9^-Ser^10^-Lxx^11^-Aib^12^-Pro^13^-Lxx^14^-Aib^15^-Lxx^16^-[C^129^]^17^
**TA-17S-III b (117)**	Ac-Aib^1^-Ala^2^-Aib^3^-Ala^4^-Vxx^5^-Gln^6^-Aib^7^-Aib^8^-Aib^9^-Ser^10^-Lxx^11^-Aib^12^-Pro^13^-Lxx^14^-Aib^15^-Lxx^16^-[C^129^]^17^
**TA-17S-III c (118)**	Ac-Ala^1^-Aib^2^-Ala^3^-Aib^4^-Vxx^5^-Gln^6^-Aib^7^-Aib^8^-Aib^9^-Ser^10^-Lxx^11^-Aib^12^-Pro^13^-Lxx^14^-Aib^15^-Lxx^16^-[C^129^]^17^

**Table 6:** Sequences of trichorzianines (**119**–**122**). The notations Vxx and Lxx indicate positions where Val/Iva and Ile/Leu are exchangeable. The superscript (apex) indicates the position of the residue within the sequence.

Trichorzianines (TA)	Sequence
**TA-19A-I a (119)**	Ac-Aib^1^-Ala^2^-Ala^3^-Aib^4^-Aib^5^-Gln^6^-Aib^7^-Aib^8^-Aib^9^-Ala^10^-Lxx^11^-Aib^12^-Pro^13^-Vxx^14^-Aib^15^-Lxx^16^-Gln^17^-Gln^18^-Pheol^19^
**TA-19A-II a (120)**	Ac-Aib^1^-Ala^2^-Ala^3^-Aib^4^-Vxx^5^-Gln^6^-Aib^7^-Aib^8^-Aib^9^-Ala^10^-Lxx^11^-Aib^12^-Pro^13^-Vxx^14^-Aib^15^-Lxx^16^-Gln^17^-Gln^18^- Pheol^19^
**TA-19A-III a (121)**	Ac-Aib^1^-Ala^2^-Ala^3^-Aib^4^-Vxx^5^-Gln^6^-Aib^7^-Aib^8^-Aib^9^-Ala^10^-Lxx^11^-Aib^12^-Pro^13^-Lxx^14^-Aib^15^-Lxx^16^-Gln^17^-Gln^18^- Pheol^19^
**TA-19 S-I a^a^) (122)**	Ac-Aib^1^-Ala^2^-Ala^3^-Aib^4^-Aib^5^-Gln^6^-Aib^7^-Aib^8^-Aib^9^-Ser^10^-Lxx^11^-Aib^12^-Pro^13^-Lxx^14^-Aib^15^-Lxx^16^-Gln^17^-Gln^18^- Pheol^19^

**Table 7:** Peptaibols from marine-derived fungi described in the paper.

Name of Compound	N.	Isolated from	Marine-Derived Fungi	Biological Activity	Ref.
Trichobrachin A-I-IV	**2**–**5**	*T. longibrachiatum*	*Mytilus edulis*	Cytotoxic	[68]
Trichobrachin B-I-IV	**6**–**9**	*T. longibrachiatum*	*Mytilus edulis*	Cytotoxic	[68]
Trichobrachin TV-Ib/IIA	**10**	*T. longibrachiatum*	*Mytilus edulis*	Cytotoxic	[68]
Trichobrachin C I-II	**11**–**12**	*T. longibrachiatum*	*Mytilus edulis*	Cytotoxic	[68]
**Trichobrachin A-VI a–e**	**13**–**17**	*T. longibrachiatum*	*Mytilus edulis*	Cytotoxic	[68]
Trichobrachin A-VII a–j	**18**–**27**	*T. longibrachiatum*	*Mytilus edulis*	Cytotoxic	[68]
Trichobrachin A-IV a–d	**28**–**31**	*T. longibrachiatum*	*Mytilus edulis*	Cytotoxic	[68]
**Trichobrachin A-VIII a–e**	**32**–**36**	*T. longibrachiatum*	*Mytilus edulis*	Cytotoxic	[68]
Trichobrachin A-IX	**37**	*T. longibrachiatum*	*Mytilus edulis*	Cytotoxic	[68]
Longibrachin A-I	**38**	*T. longibrachiatum* *(T. koningii)*	Several sources	Antimicrobial, cytotoxic, genotoxic	[68]
Longibrachin A-0	**40**	*T. longibrachiatum * (MMS151)	*Mytilus edulis*	Cytotoxic, antibacterial, antifungal	[73]
Longibrachin A-II-a	**41**	*T. longibrachiatum * (MMS151)	*Mytilus edulis*	Cytotoxic, antibacterial, antifungal	[73]
Longibrachin A-IV-b	**42**	*T. longibrachiatum * (MMS151)	*Mytilus edulis*	Cytotoxic, antibacterial, antifungal	[73]
Asperelines A-F	**43**–**48**	*T. longibrachiatum * *T. asperellum* (Y19-07)	Soil	Antimicrobial	[78]
Aspereline G	**49**	*T. asperellum*	Marine sediments	Cytotoxicity	[24,75]
Aspereline H	**50**	*T. asperellum*	Marine sediments	Cytotoxicity	[24,75]
Asperelines I-Z	**51**–**67**	*T. asperellum*	Marine sediments	Cytotoxicity	[24]
Asperelines Z_1_–Z_13_	**68**–**80**	*T. asperellum*	Marine sediments	Cytotoxicity	[24]
Asperelines Z_14_–Z_22_	**81**–**89**	*T. asperelloides*	*Victoria amazonica*	Antimicrobial	[76]
**Trichorzianine 17A I a–e**	**90**–**94**	*T. atroviride* (MMS927)	Shellfish	Cytotoxic	[25]
Trichorzianine 17A II a–f	**95**–**100**	*T. atroviride* (MMS927)	Shellfish	Cytotoxic	[25]
**Trichorzianine 17A III a–d**	**101**–**104**	*T. atroviride* (MMS927)	Shellfish	Cytotoxic	[25]
Trichorzianine 17A IV a	**105**	*T. atroviride* (MMS927)	Shellfish	Cytotoxic	[25]
Trichorzianine 17S I a–f	**106**–**111**	*T. atroviride* (MMS927)	Shellfish	Cytotoxic	[25]
Trichorzianine 17S II a–d	**112**–**115**	*T. atroviride* (MMS927)	Shellfish	Cytotoxic	[25]
Trichorzianine 17S III a–c	**116**–**118**	*T. atroviride* (MMS927)	Shellfish	Cytotoxic	[25]
Trichorzianine 19A I a	**119**	*T. atroviride* MMS927	Shellfish	Cytotoxic	[25]
Trichorzianine 19A II a	**120**	*T. atroviride* (MMS927)	Shellfish	Cytotoxic	[25]
Trichorzianine 19A III a	**121**	*T. atroviride* (MMS927)	Shellfish	Cytotoxic	[25]
Trichorzianine 19S I a	**122**	*T. atroviride* (MMS927)	Shellfish	Cytotoxic	[25]
Trichorzianine 1938	**123**	*T. atroviride* (NF16)	Sponge	Antimicrobial	[77]
Trichorzianine 1909	**124**	*T. atroviride* (NF16)	Sponge	Antimicrobial	[77]
Trichorzianine 1895	**125**	*T. atroviride* (NF16)	Sponge	Antimicrobial	[77]
Trichorzianine 1896	**126**	*T. atroviride* (NF16)	Sponge	Antimicrobial	[77]
Trichorzianine 1924	**127**	*T. atroviride* (NF16)	Sponge	Antimicrobial	[77]
Trichorzianine 1910	**128**	*T. atroviride* (NF16)	Sponge	Antimicrobial	[77]
Trichorzianine 1924a	**129**	*T. atroviride* (NF16)	Sponge	Antimicrobial	[77]
Trichorzianine 1909a	**130**	*T. atroviride* (NF16)	Sponge	Antimicrobial	[77]
Trichorzianine TA-VI b	**131**	*T. atroviride* (NF16)	Sponge	Antimicrobial	[77]
Trichorzianine TA-VI A	**132**	*T. atroviride* (NF16)	Sponge	Antimicrobial	[77]
Trichorzianine TA-VII	**133**	*T. atroviride* (NF16)	Sponge	-	[77]
Trichorzianine TA-V b	**134**	*T. atroviride* (NF16)	Sponge	Antimicrobial	[77]
Hyporientalin A	**135**	*T.* *orientale*	*Cymbaxinella damicornis*	Antibacterial, antifungal	[79]
Pentadecaibins I–V	**136**–**140**	*T. harzianum (MMS1255)*	Sediment	Antimicrobial, cytotoxic	[61]
Trichorzins A–G	**141**–**147**	*Trichoderma* sp. (GXIMD 01001)	*Haliclona* sp	Cytotoxic	[86]
Lipotrichaibol A	**148**	*Trichoderma* sp. (GXIMD 01001)	Sponge	Antiproliferative, cytotoxic	[88]
Trichoderpeptides A–D	**149**–**152**	*Trichoderma* sp. (GXIMD 01001)	Sponge	-	[88]
Emerimicin IV	**153**	*Emericellopsis minima*	Sediment	Bacteriostatic	[89]
Emericellipsin A	**154**	*Emericellopsis alkalina*	Several sources	Cytotoxic	[90]
RHM1	**155**	*Acremonium* sp. (021172cKZ)	*Teichaxinella* sp.	Cytotoxic, antibacterial	[92]
RHM2	**156**	*Acremonium* sp. (021172cKZ)	*Teichaxinella* sp.	Cytotoxic	[92]
Efrapeptin G	**157**	*Acremonium* sp. (021172cKZ)	*Teichaxinella* sp.	Cytotoxic	[92,94]
Acremopeptaibols A–F	**158**–**163**	*Acremonium* sp. (IMB18-086)	*Haliclona* sp.	Antimicrobial, antifungal	[100]
Microbacterins A–B	**164**–**165**	*Microbacterium sediminis* spp. YLB-01-T	Sediment	Cytotoxic	[29]
Tolypocaibols A–B	**166**–**167**	*Tolypocladium* sp. (KP1-175E)	*Spongomorpha arcta*	Antibacterial	[101]
SK-P1–SK-P6	**168**–**173**	*Stephanonectria keithii* (LZD-10-1)	*Peseudopterogorgia* sp. LZD-10	Antibacterial	[102]

## Data Availability

The original contributions presented in this study are included in the article. Further inquiries can be directed to the corresponding author.
